# A heat-melt adhesive-assisted transferable electrode films

**DOI:** 10.1038/s41598-020-79504-7

**Published:** 2021-01-08

**Authors:** Yuki Maruyama, Kuniaki Nagamine, Shigeyuki Iwasa, Atsushi Miyabo, Shizuo Tokito

**Affiliations:** 1 Department of Organic Materials Science, Yamagata University, Yamagata, Japan; 2 Research Center for Organic Electronics, Yamagata University, Yamagata, Japan; 3 Arkema K. K., 2-2-2 Uchisaiwaicho, Chiyoda-ku, Tokyo, 100-0011 Japan

**Keywords:** Materials for devices, Electrical and electronic engineering

## Abstract

This report is the first on heat-assisted transferable battery components, enabling manufacturing batteries on non-planer surfaces such as a curved surface and an edge. The transferrable battery components were composed of two layers: a cathode or an anode and a conductive heal-melt adhesive layer on a silicone-based flexible supporting paper. These mechanically-durable, flexible components enabled conformable adhesion even on curved surfaces and substrate edges. As a model battery, the manganese dioxide-zinc system was constructed on a curved surface using transfer techniques and showed a practical capacity of 1.8 mAh cm^−2^ per unit electrode area. These transferable electrodes allow arbitrary design of batteries according to the power consumption of IoT devices to be fabricated on unreported geometries where has been considered as a dead space.

## Introduction

Integrating various kinds of sensors on the human body or products, and public facilities are required to enable the emerging Internet of Things (IoT) society. Recently developed wearable sensors with flexible, bendable, and stretchable characteristics are designed to be conformable to these objects with complex surface geometry. For example, wearable health sensing devices are worn on the human body in the form of wristband, band-air, or soft contact lens^1–5^. For logistics tracking applications, the product tag with an environmental sensor has been developed to be placed on products^6^. The other compelling application is a wearable device for plants to monitor their growth and surrounding environmental conditions^7^. Whatever the sensor's application, an energy storage device is required for these sensors' continuous operation. Storage devices such as lithium-ion batteries and redox capacitors are suitable for repeated use, while primary batteries are expected to be installed in low-cost devices for disposable use^8–10^. As described above, the ideal mechanical characteristics IoT devices with a power supply should be conformable to the shapes of objects while not interfering with their functions and operations.

For this reason, various kinds of flexible or bendable batteries packed in a laminate film have been developed^11–13^. These batteries can be attached to objects with irregular surface structures while following their motion. However, the connection method remains an issue because the electronic circuit and the battery must be manufactured separately. Another advanced technique is a direct printing of the battery pattern on a curved surface of objects using a dispenser printing^5^. Previous research has demonstrated a redox capacitor's construction on a contact lens's curved surface by using a dispenser. This technique is useful for the additive manufacturing of an arbitrarily designed battery connecting to electronic circuits. However, this technique is challenging to scale up because the nozzle diameter restricted the electrode's width, and it needs to be repeated dozens of times. We focused on the transferring method, intending to construct electrodes large enough for the curved surface^11,14^.

This method provides easy transfer of the patterns previously fabricated on the other supporting substrate to the target objects on demand. With this method, the supporting substrate's transferrable patterns were attached to the target surface. However, the previously reported methods used a rigid support substrate such as mica or silicon wafer; the composite could not be deformed to fit the target surface geometry. Furthermore, because the transferring method was based on the object's adhesion, the transferable material was restricted to sticky materials such as poly(dimethylsiloxane).

**Figure 1:**
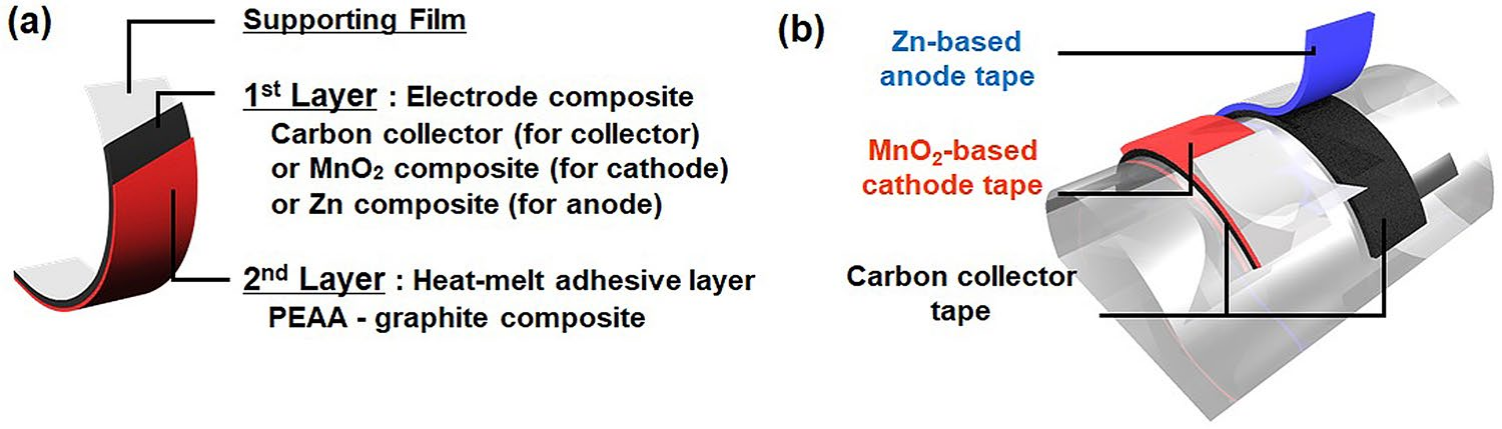
Illustration of the layered structure of transferable flexible battery components (**a**) and its application to the curved surface (**b**). These illustrations were designed with the free software POV-Ray for windows—version 3.7.0.

In this study, we developed the transferrable battery components, including carbon collector, cathode, and anode that consisted of two layers: an electrode composite layer and a conductive adhesive layer on a flexible supporting film (Fig. 1). A mechanically-durable, silicone-based flexible paper was used as a supporting film because an oxygen-plasma treatment could easily modify its surface wettability. Furthermore, the surface roughened by plasma irradiation is expected to improve adhesion to the electrode composite by the anchor effect. The conductive adhesive layer was composed of poly(ethylene-co-acrylic acid) (PEAA) as a heat-melt adhesive resin and a graphite powder as an electric conductor. The battery components can be transferred to the target surface by applying heat from the supporting film's backside. Due to the low melting point of PEAA at 70 °C, the battery components and target surface with heat-sensitive electronic circuits will receive less heat damage. A MnO_2_/Zn primary battery was constructed as a model to demonstrate the applicability of these transferable battery components and its battery characteristics.

## Results and discussion

Figure 1a illustrates the layered structure of transferable flexible battery components for the current collector and anode/cathode. They consisted of two composite layers: an electrode composite (first layer) and a heat-melt adhesive and conductive composite (second layer). The first layer's composition was as follows: (Carbon collector composite) carbon nanomaterials as conductors and polyvinylidene difluoride (PVDF) as a binder. (Cathode and anode composite) active materials (cathode: manganese dioxide, anode: zinc), multiwalled carbon nanotube (MWCNT), polystyrene-polybutadiene block copolymer as a binder, and hydroxyethyl cellulose as a thickener. The second adhesive and conductive layer was composed of graphite as a conductor and PEAA as a heat-melt adhesive resin. Slurries of the first and second layers were sequentially coated and dried on a silicone-based supporting paper. The supporting paper's surface wettability was tuned by oxygen plasma treatment for the first layer to stick to the silicone sheet^15,16^. The silicone-based paper has high durability and flexibility to be conformable to the target surface. Also, its thermal resistivity (under 250 °C) allows the heating process at 100 °C during the electrode composites' preparation on the supporting paper^17^. These transferrable battery components were placed on the target surface so that the first layer contacted the target surface and heated over 70 °C to melt the second adhesive layer. After heating, only the electrode composites were left on the target surface by peeling the supporting paper from the first layer (Fig. 1b).

First, the cathode and anode slurries were prepared by the previously reported optimized process^9^, and the carbon collector slurry was originally prepared. Cathode and anode slurries were prepared by mixing 5% MWCNT, 2.5% polystyrene-polybutadiene block copolymer and 2.5% hydroxyethyl cellulose, with 90 wt% active materials (manganese dioxide powder for the cathode and zinc for the anode) in *N*-methyl-2-pyrrolidone (NMP). In the carbon collector case, it has been reported that blending multiple carbon materials resulted in lower resistance than using single carbon material. For example, Song et al. showed that low-resistance flexible conductive films could be made by mixing MWCNT with carbon black (CB)^18^. Our study found a carbon blend composed of the vapor-grown carbon fiber (VGCF), and CB was evaluated. VGCF is one of the graphitized carbon fibers that could be homogeneously dispersed in the electrode composite layer to form a network structure, decreasing the resistance and increasing the mechanical strength^19,20^.

**Figure 2:**
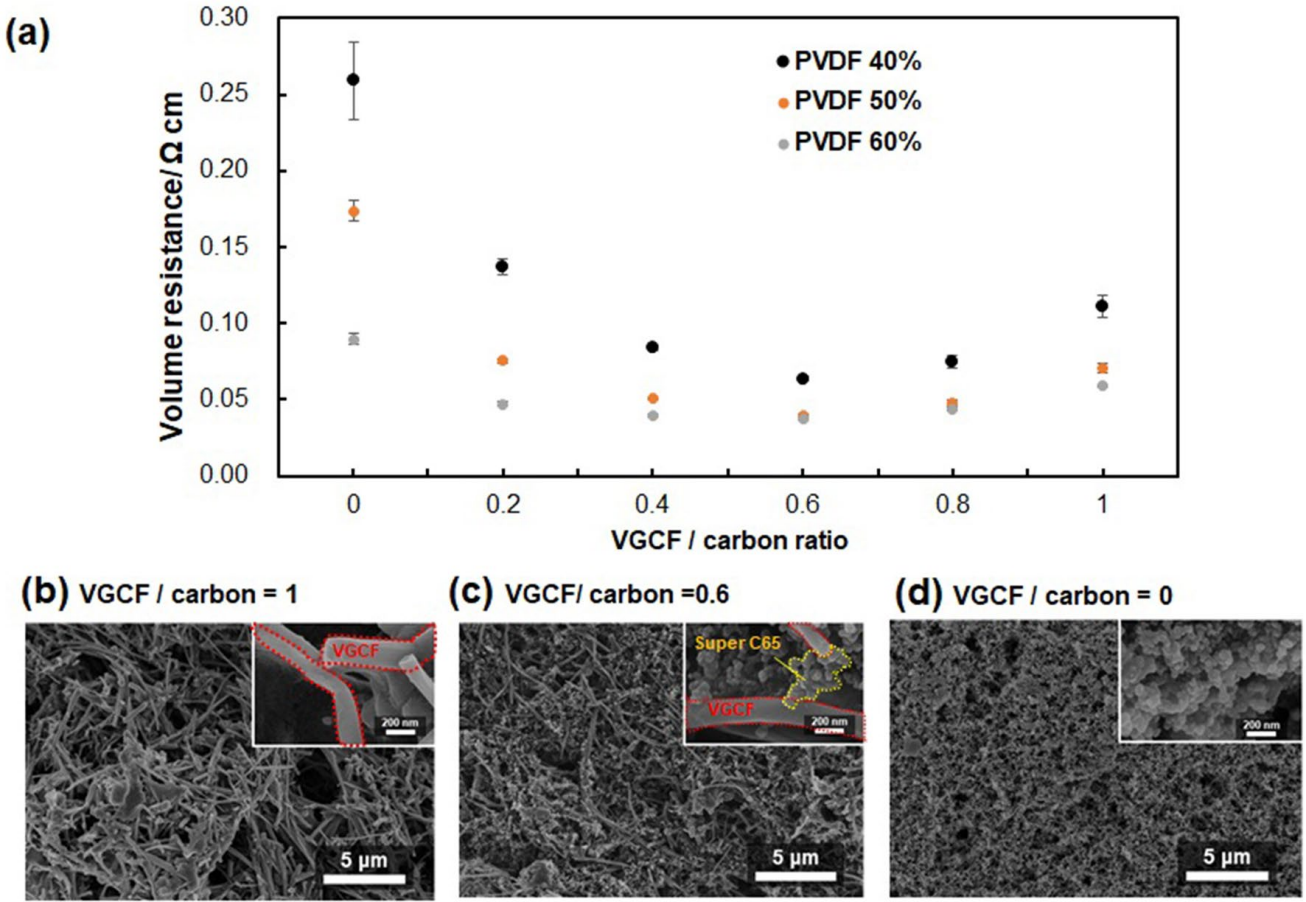
Optimization of the carbon collector composite slurry. (**a**) The carbon collector's volume resistance against VGCF/carbon ratio when PVDF binder was blended 40, 50, and 60% against the slurry's total weight. SEM images of the composites with VGCF/carbon ratio were (**b**) 1, (**c**) 0.6, and (**d**) 0 in the case of the PVDF ratio was 50%. The insets are high-magnification SEM images corresponding to each carbon composite.

The relationship between the mixture ratio of VGCF/CB/PVDF in the slurry and the volume resistance was investigated to minimize the resistance. VGCF/CB/PVDF powders (total amount of 0.2 g) were mixed with 1 g of NMP solvent, and the slurry was blade coated on a glass substrate and dried at 100 °C. The volume resistance was calculated by multiplying the sheet resistance by the composite thickness. Figure 2a shows the volume resistance of the carbon collector prepared from the various slurry compositions. The horizontal axis refers to the percentage of VGCF in carbon weight (VGCF/carbon ratio). For example, VGCF/carbon ratio = 0.6 and PVDF 50% means that 0.06 g VGCF and 0.04 g CB and 0.1 g PVDF (50% against 0.2 g of total weight of the slurry) were mixed. For the same value of VGCF/carbon ratio, the volume resistance of carbon composites decreased with an increase of the PVDF content ratio. This is because the increased PVDF binder strongly facilitated the bonding between VGCF and CB to reduce the contact resistance between them. Figure 2b–d shows the SEM images of carbon composites when the VGCF/carbon ratio was 0, 0.6, and 1. Without CB (VGCF/carbon ratio = 1), the VGCF fibers formed a mesh-like structure (Fig. 2b). In contrast, without VGCF (VGCF/carbon ratio = 0), the CB particles densely presented (Fig. 2d). When VGCF/carbon ratio was 0.6, VGCF fiber formed a mesh-like network connected via CB particles (Fig. 2c). These results suggested an increased number of contact points between VGFC fibers via CB particles decreased the bulk volume resistance of the carbon collector composite. In the following study, the carbon collector slurry composition was set to VGCF/carbon ratio = 0.6, PVDF content ratio = 50%.

**Figure 3:**
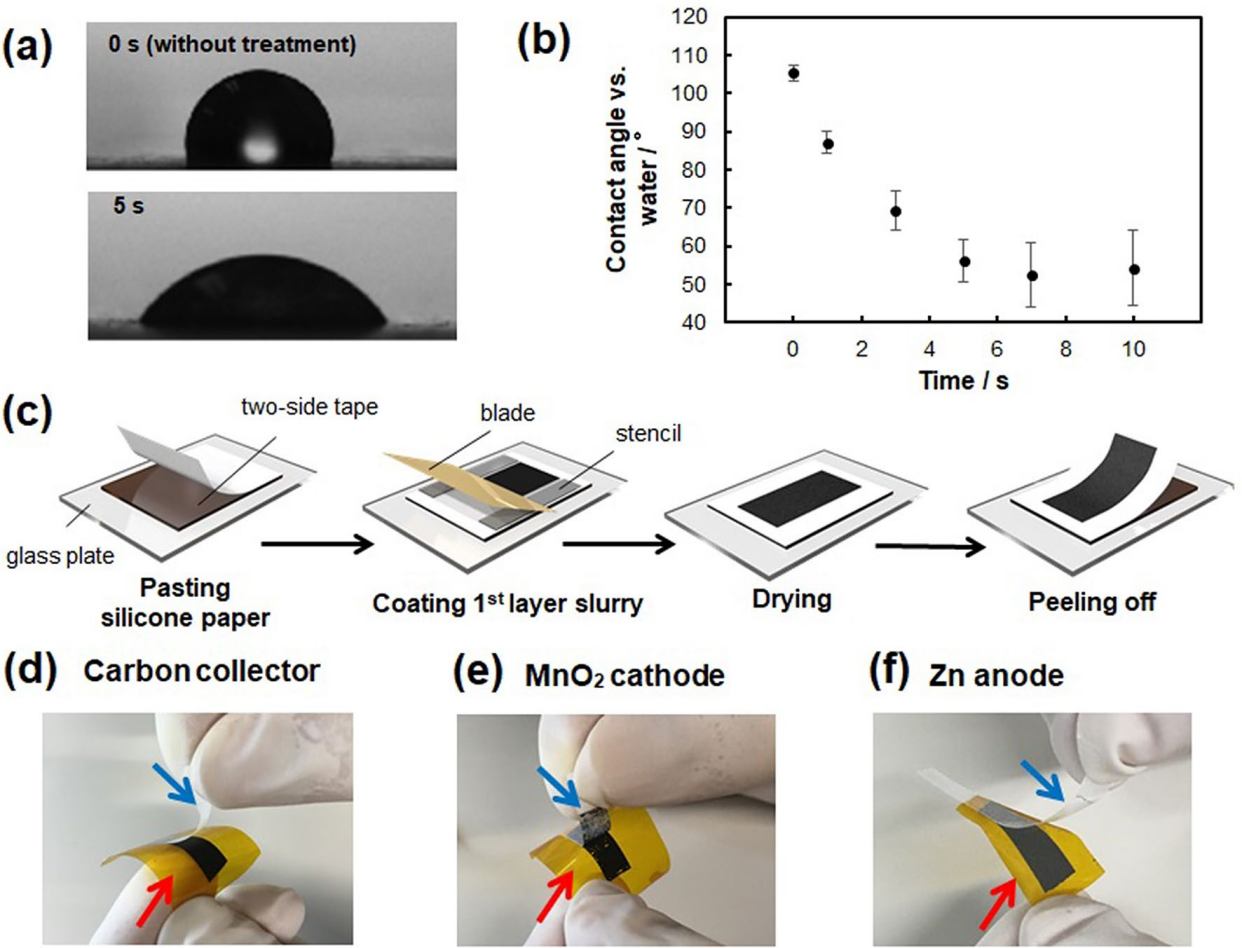
Oxygen plasma treatment of the silicone-based supporting paper surface with oxygen plasma and preparation of first layer composites. (**a**) Water droplets on the pristine and oxygen plasma-treated (100 W for 5 s) silicone-based paper. (**b**) Contact angles of water droplets on the supporting paper treated for different irradiation time. (**c**) Steps for the preparation of the first layer composite on the oxygen-plasma-treated paper. (**d**–**f**) Photographs of the peeled Kapton tape (red arrow) with the electrode composite from the plasma-treated silicone-based paper (blue arrow). The illustration of (**c**) designed with the free software POV-Ray for windows—version 3.7.0.

As a preliminary study of the silicone-based supporting paper's surface modification, the effect of oxygen plasma treatment on the supporting paper's wettability against water was evaluated using the contact angle measurement. Figure 3a shows water droplets' appearance on the pristine and oxygen plasma-treated (100 W for 5 s) silicone-based paper. The contact angle decreased after oxygen plasma treatment. Figure 3b plots the water droplet's contact angle on the paper for the oxygen plasma irradiation time. The contact angle decreased in a time-dependent manner within 5 s of irradiation and approached a constant value at more than 5 s.

Then, adhesion of the first layers (carbon collector, cathode, and anode composites) to the oxygen plasma-treated silicone-based supporting papers was evaluated. Each electrode composite was prepared on the oxygen plasma-treated paper by blade coating with 125 µm thick stencils and then drying (Fig. 3c). Then, adhesive Kapton tape was attached to the electrode composite, followed by peeling the supporting paper's tape. Figure 3d–f show the peeled Kapton tape (orange-color tape pointed by the red arrow) with the electrode composite from the supporting paper (white-color paper pointed by the blue arrow). All the electrode composites were successfully peeled from the oxygen plasma-treated supporting paper. Supplementary Figure S1 shows photographs of the peeled Kapton tape from the supporting paper treated for different oxygen plasma irradiation time (1, 3, and 5 s). The collector electrode and cathode composites were spontaneously detached from the paper treated for less than threshold irradiation time (3 s for the collector electrode, 1 s for the cathode, respectively) without Kapton tape due to its weak adhesion to the paper (Supplementary Figure S2). Optimal adhesion of the carbon collector composite was achieved by treating the paper for 5 s, and it was successfully transferred onto Kapton tape. The relatively brittle manganese dioxide layer was partially destroyed during transferring. The weight of the transferred manganese dioxide composite and the remaining composite on the silicone sheet were measured and the residual ratio was calculated to be 5% for 3 s-treated paper. As the irradiation time increased to 5 s, the residual ratio increased to 8%, so that the optimal irradiation time was determined to be 3 s.

On the other hand, anode composite showed good adhesion even on the 1 s-treated paper, while its adhesion against 5 s-treated paper was too strong to be peeled from the paper. Based on these results, the optimum oxygen plasma irradiation time against the silicone-based papers for the carbon collector, cathode, and anode composites was determined to be 5, 3, and 1 s, respectively.

**Figure 4:**
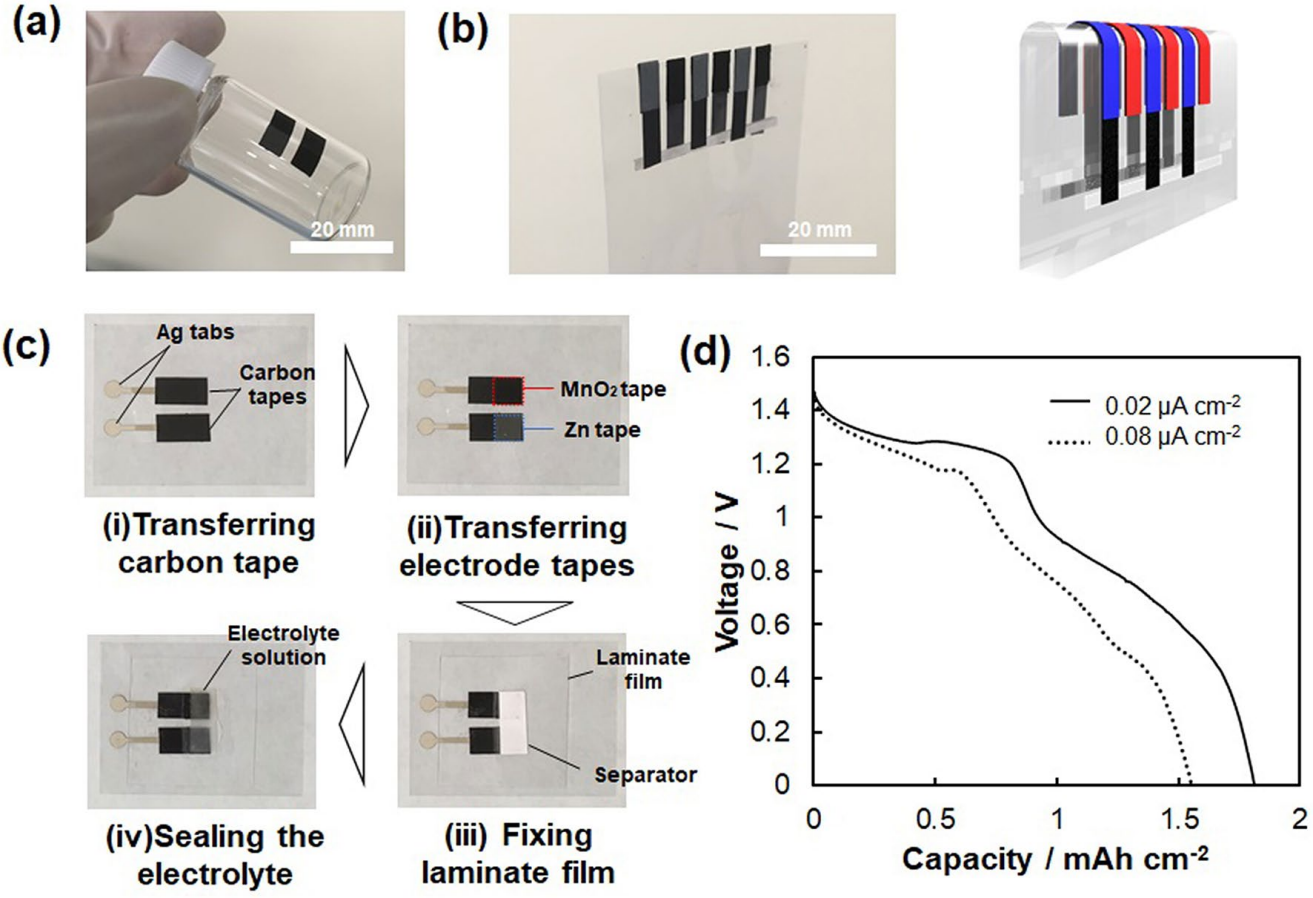
(**a**,**b**) Photograph of the transferred battery components onto the curved surface of a glass bottle (**a**) and the edge of PEN film (**b**). (**c**) The transfer-based fabrication process of the primary battery. (**d**) Discharging profile of the primary battery at different speeds of 0.02 and 0.08 µA cm^−2^. The illustration in (**b**) was designed with the free software POV-Ray for windows—version 3.7.0.

Next, the second adhesive/conductive layer was formed on the first layer-prepared supporting paper. The slurry consisting of graphite and PEAA was prepared into α-terpineol solvent, followed by blade-coating on the first layer. Finally, α-terpineol was removed by vacuum drying at 5 °C to prevent the solvent from soaking into the first layer. Supplementary Figure S3 shows the PEAA-graphite layers' cross-sectional profiles and SEM images on the manganese dioxide cathode (a,c) and zinc anode layers (b,d). The cathode and anode composites' thicknesses before and after the second layer coating were 81 to 89 µm and 49 to 57 µm, respectively, suggesting that the second layer's thickness was about 8 µm. The SEM image of the PEAA-graphite layer on first layer (manganese dioxide and zinc layer) showed a clear interface between the two layers. The blend of the two layers was prevented. Figure 4a shows the demonstration of the transfer of the battery components onto a curved surface. Two carbon collectors were adhered in parallel onto the curved sidewall of a glass bottle and then heated using the iron. Then, the silicone-based supporting paper was peeled off from the composites. Figure 4b shows another demonstration of battery components transferred to the steep edge of the polyethylene naphthalete (PEN) film (50 µm thick). Two pairs of silver wire patterns were formed from the front surface to the PEN film's back surface, and the cathode/carbon collector was transferred onto one of the silver wires and the anode/carbon collector to the other. If the electrolyte solution was sealed on the electrode surface, it could function as a battery. In general, the edge of the film was unsuitable for forming devices. Our technique enables the construction of a battery even at such dead space to effectively use the objects' limited area.

Finally, the primary battery was constructed by transferring carbon collector, cathode, and anode composite onto a PEN film, a commonly used substrate for printed electronics. Figure 4c shows the transfer-based fabrication process of the primary battery. 5 mm width of carbon collector composites were transferred on a pair of Ag tab electrode patterns previously prepared on the PEN film in parallel at 2 mm intervals. The cathode and anode tape cut into 5 mm squares were transferred to the carbon collector's tip to prepare the anode and cathode electrode (1). A transparent laminate film was heat-fused to one side of the Ag tab (2). A paper separator was placed between the laminate film and the anode and cathode electrodes, followed by applying 12 µL (14 mg) of electrolyte (NH_4_Cl 20 wt% + ZnCl_2_ 20 wt%) into it (3). Finally, the three sides of the laminate film were thermally fused to seal the electrolyte (4). Figure 4d shows the results of the discharge test of the fabricated primary battery. The plateau behavior was observed around 1.3 and 0.9 V, respectively. The first plateau region at 1.3 V is due to the reaction in which MnO_2_ is reduced to MnO(OH). The second step is derived from the reduction of MnO(OH) to form Mn^2+^ or Mn_3_O_4_. This behavior is observed in the case that MWCNT was used as conductive carbon^9^. Through this two-step reaction, the electrode tape exerted a capacity of 1.8 mAh cm^−2^.

## Conclusions

We developed heat-assisted transferable battery components to achieve the battery's arbitral design, which is compatible with IoT devices. We found that cost-effective silicone-based paper was suitable as a supporting substrate for the transfer technique because its surface wettability could be tuned by an oxygen plasma treatment depending on the kinds of electrode composites. A primary MnO_2_/Zn battery consisting of three types of transferable electrodes (carbon collector, cathode, and anode) demonstrated a capacity of 1.8 mAh cm^−2^. Since this novel transferrable battery components can be designed to any shape, it is expected that the battery shape, which is calculated backward from the power consumption of the device, can be produced readily. This technique will also make it possible to construct the battery on unreported geometries such as curved surfaces and edges. Besides, in contrast to printing techniques that require a printing mask, these electrodes can be designed arbitrarily by cutting. This allows for arbitral battery design according to the power consumption of various IoT devices. However, the present study remains a qualitative discussion about the transfer conditions. A quantitative discussion in light of silicone paper surface roughness and polarity should be conducted in the near future. Furthermore, to further increase the battery density, it is preferable to stack electrodes and separator/electrolyte. To achieve the sandwich structure, the development of sticky separator/electrolyte sheet and electrode that can be transferred to the electrolyte sheet is the next challenges.

## Methods

### Materials

Polystyrene-block-polybutadiene-block-polybutadiene (styrene 30%, Mw ~ 140,000), poly(ethylene-co-acrylic acid) (acrylic acid 20 wt%) and graphite powder were purchased from Sigma Aldrich. Hydroxyethyl cellulose and multiwalled carbon nanotube (10–20 nm of diameter, 5–15 µm of length) were purchased from TCI. *N*-Methyl-2-pyrrolidone, α-terpineol and zinc powder were purchased from Wako. Zinc chloride and ammonium chloride were purchased from Kanto. Manganese dioxide powder was purchased from Junsei. Volatile grown carbon fiber was purchased from Showa denko. ARKEMA kindly provided poly ethylene difluoride (Kynar HSV1800). Super C65 was purchased from IMERYS Corp. Commercially available silicone sheet was purchased from the supermarket. Poly ethylene naphthalate film (G65HA) was purchased from Dupont. All materials were employed without further purification.

### Preparation of the carbon collector/MnO_2_/Zn slurry and PEAA-graphite

Carbon slurry was prepared by uniformly mixing VGCF, Super C65 and PVDF powder, followed by adding 1 g of NMP to be mixed together using an agate mortar. Cathode slurry was prepared by mixing 0.05 g of MWCNT, 0.125 g of 20%-polystyrene-polybutadiene block copolymer/NMP solution, 0.5 g of 5%-hydroxyethyl cellulose/NMP, and 0.4 g of NMP, followed by adding 0.90 g of MnO_2_ powder to be mixed together using an agate mortar. Anode slurry was prepared by mixing 0.05 g of MWCNT was mixed along with 0.125 g of 20%-polystyrene-polybutadiene block copolymer/NMP solution, 0.5 g of 5%-hydroxyethyl cellulose/NMP, and 0.1 g of NMP, followed by adding 0.90 g of Zn powder to be mixed together using an agate mortar. PEAA-graphite slurry was prepared by mixing 2 g of 15 wt% PEAA resin in α-terpineol prepared at 65 °C with 0.3 g of graphite powder in a sample dish and heated at 50 °C. Then, the uneven slurry was stirred under cooling at 5 °C to be mixed uniformly.

### Making carbon collector/cathode/anode tape

The silicone-based paper was fixed on the glass substrate and irradiated the oxygen plasma of 100 W for arbitral time (PC-300, Samco). The first layer slurry was blade-coated on the treated silicone paper using the stencil mask (125 µm in thickness), and dried at 100 °C for 1 h in the forced airflow oven (WFO-520, EYELA). Then, the PEAA-graphite slurry was coated on the first layer using 100, 125, and 100 µm thick of stencil masks for carbon collector, cathode, and anode composite first layers, respectively. The resulting papers were cooled down to 5 °C and dried under vacuum overnight. After that, the remaining solvent was removed by drying at 65 °C overnight.

### Battery fabrication

Two Ag tab patterns were prepared by applying Ag nanoparticle paste (DOTITE, Fujikura Kasei) on the PEN substrate through a dumbbell-shaped stencil masks (10 mm for gauge length, 3 mm in diameter, in parallel at 2 mm intervals) and baked at 150 °C for 30 min. Five (5) mm wide of the carbon tapes were placed on the surface of Ag tab patterns and transferred by heating them at 75 °C; after that silicone sheets were removed from the carbon composites. PEAA/α-terpineol solution was applied to the printed Ag tabs and a dryer removed the solvent. The cathode and anode tapes cut into 5 mm squares were applied to the carbon collector's tip and transferred by heating them at 75 °C. A transparent laminate film (LZ-A4100, IRIS OHYAMA) was heat-fused to one side of the Ag tab side. A paper separator was placed between the laminate film and the electrodes, and 12 µL (14 mg) of electrolyte (NH_4_Cl 20 wt% + ZnCl_2_ 20 wt%) was injected into it. Finally, the three sides of the laminate film were thermal fusion to seal the electrolyte solution.

### Characterization

The morphology of the carbon collector was observed with a scanning electron microscope (JSM-7600FA, JEOL). The cross-sections of PEAA-graphite layer were observed with a tabletop microscope (TM4000, HITACHI). Sheet resistance was measured with the residence measurement (sigma − 5 + -S.P., NPS). The thickness of the electrode was measured with the profiler (Dektak XT, ULVAC). The contact angle was measured with the contact angle meter (Theta T200-Basic, Biolin Scientific). The discharging test was conducted with charge/discharge tester (EP-7100P, Electrofield).

## Supplementary Information

Supplementary Figures.

## Data Availability

All data generated or analyzed during this study are included in this published article and its Supplementary Information files.

## References

[1] Ostfeld AE, Gaikwad AM, Khan Y, Arias AC (2016). High-performance flexible energy storage and harvesting system for wearable electronics. Sci. Rep..

[2] Gao W, Emaminejad S (2016). Fully integrated wearable sensor arrays for multiplexed in situ perspiration analysis. Nature.

[3] Rose DP, Heikenfeld JC (2014). Adhesive RFID sensor patch to monitor sweat electrolytes. IEEE Trans. Biomed. Eng..

[4] Khan Y, Thielens A, Muin S, Ting J, Baumbauer C, Arias AC (2020). A new frontier of printed electronics: flexible hybrid electronics. Adv. Mater..

[5] Park J, Ahn DB, Kim J, Cha E, Bae BS, Lee SY, Park JU (2019). Printing of wirelessly rechargeable solid-state supercapacitors for soft, smart contact lenses with continuous operations. Sci. Adv..

[6] Quintero AV, Briand D (2016). Smart RFID label with a printed multisensor platform for environmental monitoring. Flexible Print. Electron..

[7] Zhao Y, Huang X (2019). Multifunctional stretchable sensors for continuous monitoring of long-term leaf physiology and microclimate. ACS Omega.

[8] Winter M, Brodd RJ (2004). What are batteries, fuel cells, and supercapacitors?. Chem. Rev..

[9] Madej E, Espig M, Baumann RR, Schuhmann W, La Mantia F (2014). Optimization of primary printed batteries based on Zn/MnO2. J. Power Sources.

[10] Wang Z, Bramnik N, Roy S, Di Benedetto G, Zunino JL, Mitra S (2013). Flexible zinc–carbon batteries with multiwalled carbon nanotube/conductive polymer cathode matrix. J. Power Sources.

[11] Koo M (2012). Bendable inorganic thin-film battery for fully flexible electronic systems. Nano Lett..

[12] Qian G (2018). Bioinspired, spine-like, flexible, rechargeable lithium-ion batteries with high energy density. Adv. Mater..

[13] Liao X (2019). High-energy-density foldable battery enabled by zigzag-like design. Adv. Energy Mater..

[14] Yang Y, Jeong S, Hu L, Wu H, Lee SW, Cui Y (2011). Transparent lithium-ion batteries. Proc. Natl. Acad. Sci. USA.

[15] Bodas D, Rauch JY, Khan-Malek C (2008). Surface modification and aging studies of addition-curing silicone rubbers by oxygen plasma. Eur. Polym. J..

[16] Hillborg H, Ankner JF, Gedde UW, Smith GD, Yasuda HK, Wikström K (2000). Crosslinked polydimethylsiloxane exposed to oxygen plasma studied by neutron reflectometry and other surface specific techniques. Polymer.

[17] Rajendra V, Sicard C, Brennan JD, Brook MA (2014). Printing silicone-based hydrophobic barriers on paper for microfluidic assays using low-cost ink jet printers. Analyst.

[18] Song WJ (2018). Jabuticaba-inspired hybrid carbon filler/polymer electrode for use in highly stretchable aqueous li-ion batteries. Adv. Energy Mater..

[19] Wu MS, Lee JT, Chiang PCJ, Lin JC (2007). Carbon-nanofiber composite electrodes for thin and flexible lithium-ion batteries. J. Mater. Sci..

[20] Miyagawa H, Misra M, Mohanty AK (2005). Mechanical properties of carbon nanotubes and their polymer nanocomposites. J. Nanosci. Nanotechnol..

