# Supplementary material for: A heat-melt adhesive-assisted transferable electrode films
Source: Sci Rep. 2021 Jan 8;11:36. doi: 10.1038/s41598-020-79504-7 (PMC7794357; doi:10.1038/s41598-020-79504-7)
Supplement: Supplementary file 1 — Supplementary Figures. [file 41598_2020_79504_MOESM1_ESM.pdf]

Supporting Information

A Heat-Melt Adhesive-Assisted Transferable Electrode Films

*Yuki Maruyama, Kuniaki Nagamine *, Shigeyuki Iwasa, Atsushi Miyabo and Shizuo Tokito**

Research Center for Organic Electronics, Yamagata University,

4-3-16, Jonan, Yonezawa, Yamagata, 992-8510, Japan

E-mail; nagamine@yz.yamagata-u.ac.jp, tokito@yz.yamagata-u.ac.jp

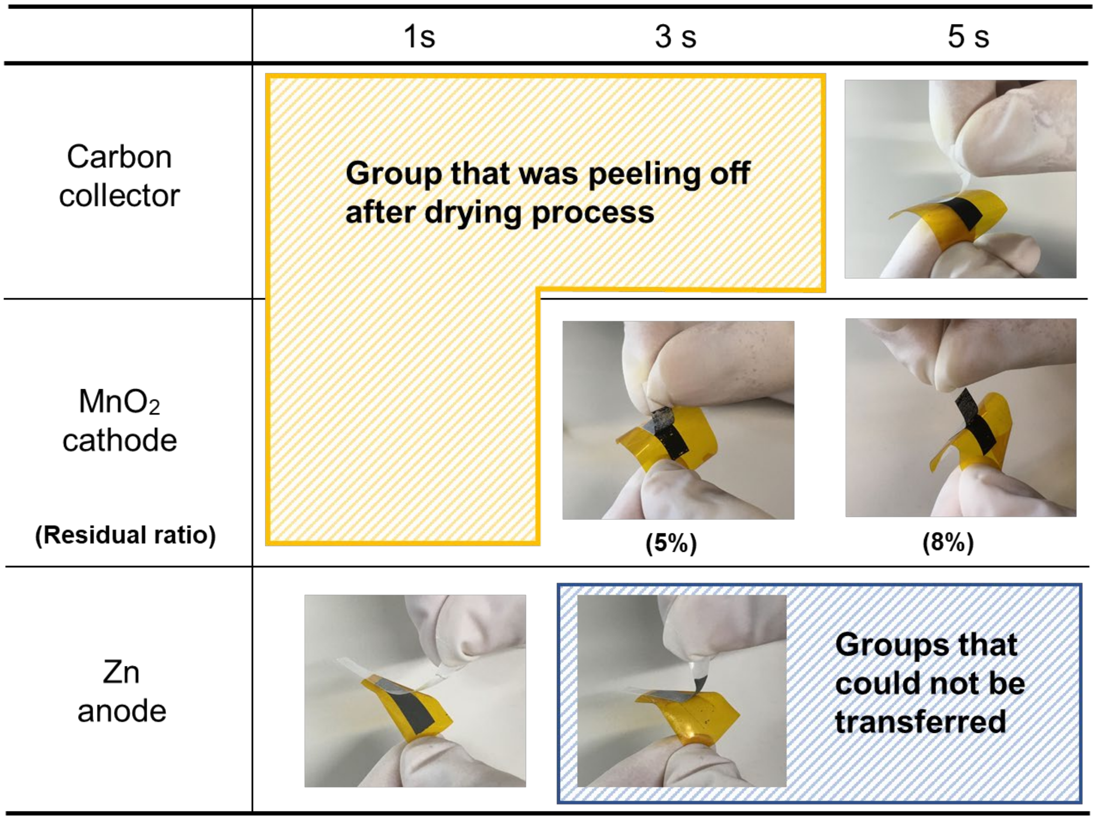

Supplementary Figure S1. Photographs the peeled Kapton tape together with carbon collector, MnO_2 cathode, and Zn anode composites from the supporting paper treated for different oxygen plasma irradiation time (1, 3, and 5 s).

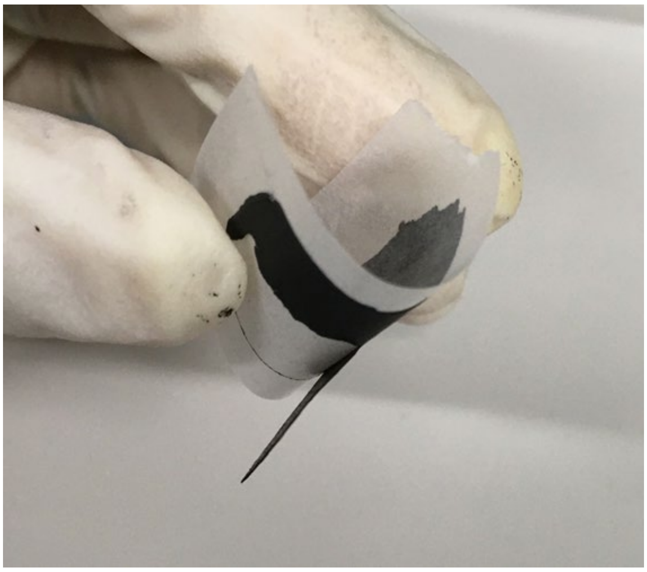

Supplementary Figure S2. A flexing view of the manganese dioxide composite on the 1 s-oxygen plasma treated silicone paper.

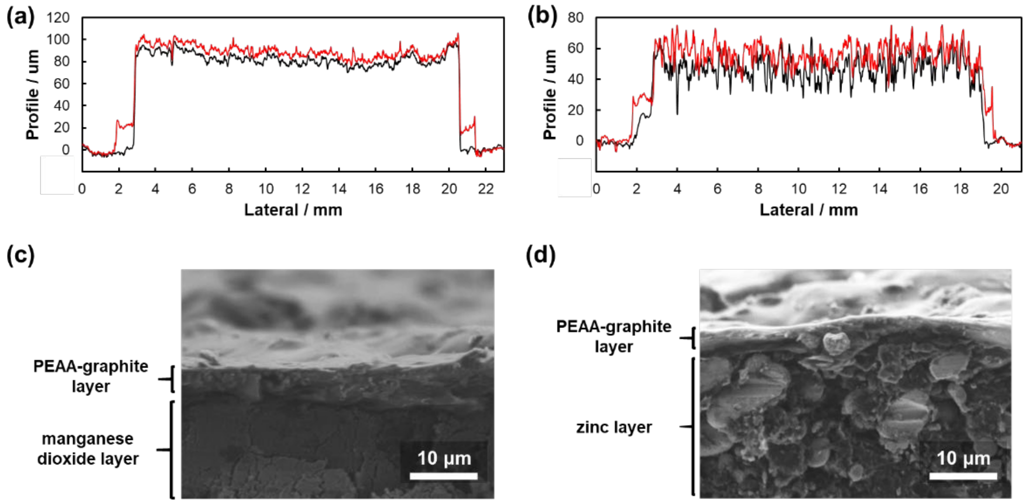

Supplementary Figure S3. The cross-sectional profiles of MnO_2 cathode(a) and zinc anode (b). The black and red lines show the profiles before (black) and after (red) formation of the graphite-PEAA 2nd layer. The PEAA-graphite layers' cross-sectional SEM images on the manganese dioxide cathode (c) and zinc anode layers (d).
